# Left Lung Collapse and Right Tension Pneumothorax: A Tale of Endobronchial Intubation and Ventilation

**DOI:** 10.7759/cureus.97491

**Published:** 2025-11-22

**Authors:** Nissar Shaikh, Maher Abu Sunbol, Malina Abualjubain

**Affiliations:** 1 Surgical Intensive Care Department, Hamad Medical Corporation, Doha, QAT; 2 Medical Education Department, Hamad Medical Corporation, Doha, QAT

**Keywords:** barotrauma, collapse, endobronchial intubation, patient movement, pneumothorax

## Abstract

Endobronchial intubation (EBI) is a relatively uncommon but well-recognized complication during anesthesia, often presenting with unexplained oxygen desaturation. Patient positioning or movement during anesthesia increases the risk for EBI. It causes lung collapse and may require intensive care therapy. The therapeutic intervention for EBI, namely, proper tube positioning and aggressive ventilation, can result in tension pneumothorax (TP), a correlation that is not reported in the literature. We report a case of EBI causing lung collapse and TP requiring unplanned admission to the intensive care unit. A young female patient was posted for craniotomy and excision of recurrent meningioma. Induction of anesthesia and intubation were uneventful. The procedure was performed in the supine position, and the patient developed oxygen desaturation at the end of surgery. It was realized by auscultation and flexible bronchoscopy that the desaturation was due to EBI and left lung collapse. The endotracheal tube was adjusted and ventilated with an Ambu bag with an improved tidal volume. Postoperatively, the patient remained in borderline oxygen saturation and developed hemodynamic instability requiring vasopressor. On chest point-of-care lung ultrasound (POCUS) examination, a right-sided pneumothorax was detected. Initially, needle decompression was done, followed by chest drain insertion. The patient was admitted to the intensive care unit (ICU), their hemodynamics gradually improved, and the chest drain was removed by day two of ICU admission. The patient was then transferred to the ward, from where he was later discharged home. EBI should be suspected in cases of perioperative desaturation. If a patient deteriorates further after tube repositioning, one should suspect pneumothorax and tension pneumothorax.

## Introduction

Endobronchial intubation (EBI) occurs in around 4% of reported incidents during anesthesia [1]. EBI is a common etiology of oxygen desaturation in the perioperative period, as it is difficult to appreciate the chest movements and may not be detected with capnography [1]. The depth of insertion may be an indicator of EBI [1,2]. It can lead to lung collapse and unplanned admission to the intensive care unit [1]. The occurrence of tension pneumothorax during the opening of the collapsed lung due to the EBI is not reported in the literature. We report a case of TP after lung collapse due to EBI.

## Case presentation

A 49-year-old female, who is 5 feet and 3 inches in height and with a body weight of 60 kg, is a known case of type 2 diabetes mellitus on oral hypoglycemic agents and recurrent meningioma. She was posted for craniotomy and excision of the tumor.

**Figure 1:**
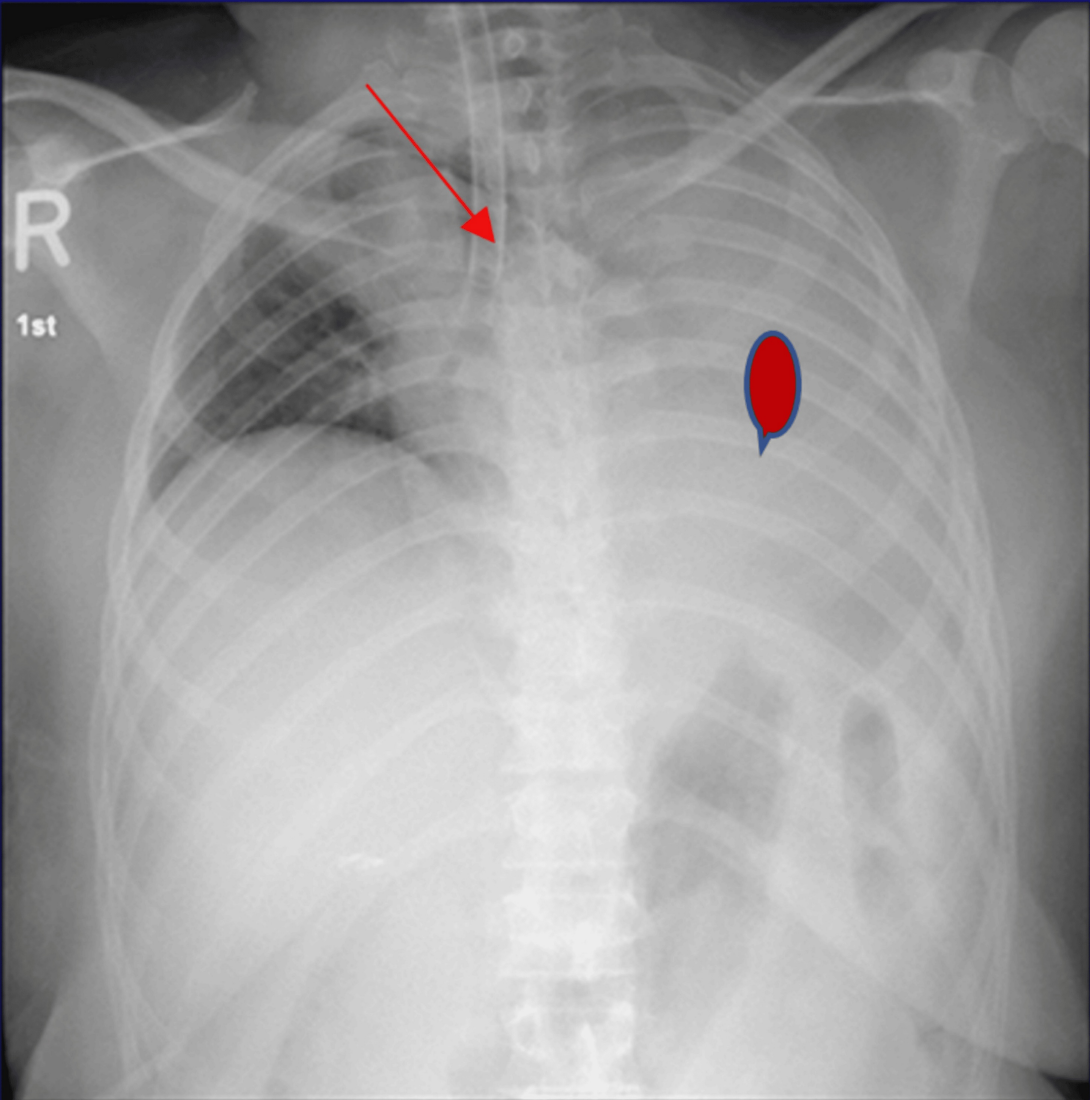
Endobronchial intubation and left lung collapse Left arrow: endotracheal tube in the right main bronchus. Right arrow: left lung collapse.

Perioperative and intraoperative periods were uneventful; however, at the end of surgery, the patient desaturated down to 70%, and her arterial blood gas (ABG) showed a PH of 7.20, PaO_2_ of 61 mmHg, PCO_2_ of 56 mmHg, and HCO_3_- of 21.1 mEq/L. Immediately, the fraction of inspired oxygen (FiO_2_) was increased to 100% with some improvement in oxygen saturation, but auscultation showed markedly decreased air entry on the left side of the chest. A portable chest X-ray revealed left lung collapse and endobronchial intubation (Figure 1). Immediate flexible bronchoscopy confirmed endobronchial intubation and a partially blocked armored tube, a tube commonly used in brain surgery. The tube was initially fixed at the 21 cm level, and at the time of desaturation, it was at the level of 23 cm. It was changed to a Portex tube and positioned in the trachea above the carina under vision with a bronchoscope. Ambu bag ventilation was performed with an improved tidal volume. She was hemodynamically stable; however, her oxygen requirement was high. She was on 100% oxygen with an oxygen saturation of 90% and high peak pressures; hence, she was transferred to the surgical intensive care unit (SICU), intubated, and sedated.

**Figure 2:**
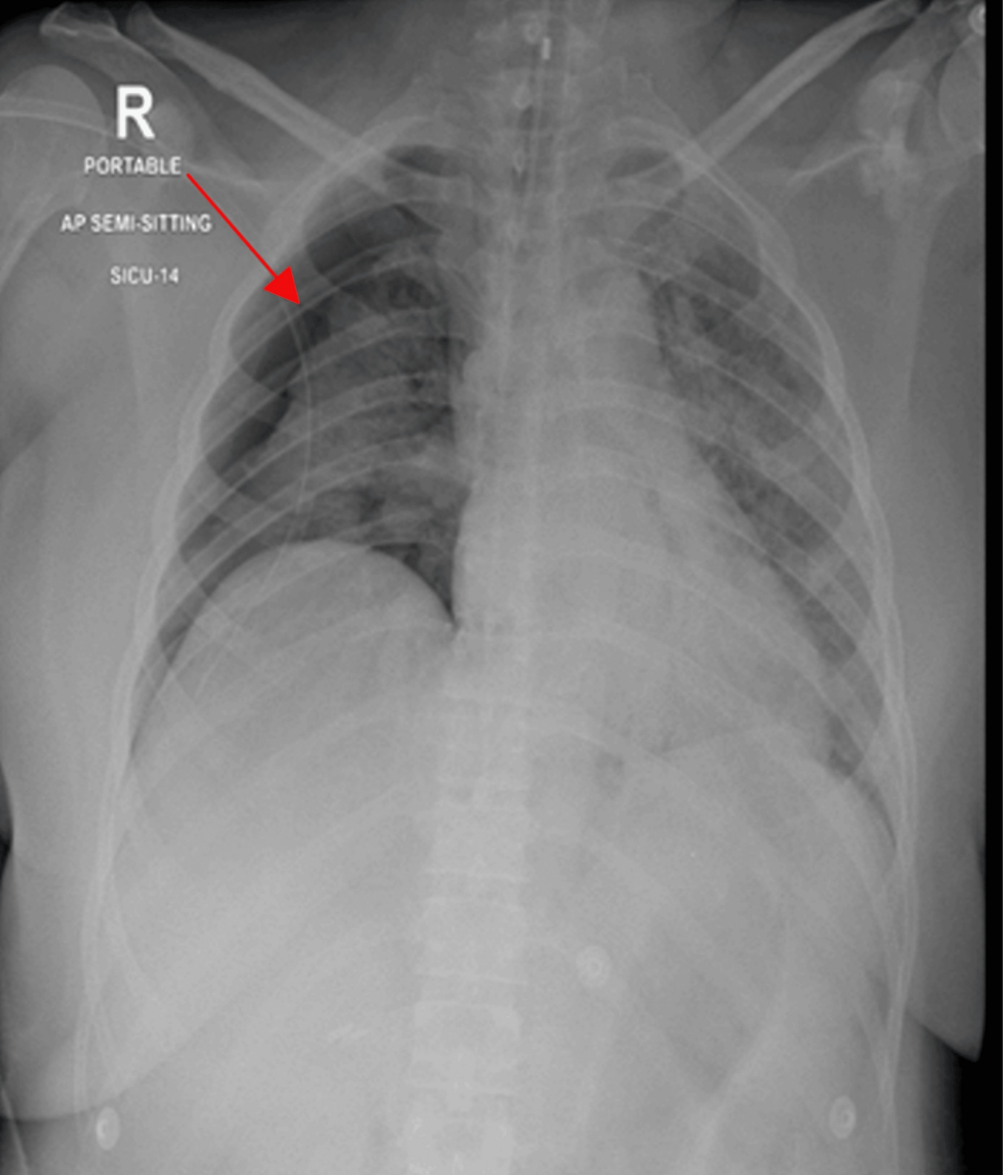
Barotrauma to the normal right lung causing pneumothorax Arrow: Right-sided pneumothorax

In the SICU, the patient was continued on mechanical ventilation, with pressure control mode. The pressure control was set at 20 cmH_2_O, positive end-expiratory pressure (PEEP) at 10 cmH₂O, and an FiO₂ of 1. On repeated chest auscultation, there was no appreciated air entry over the right chest, and on percussion, the right side of the chest was hyper-resonant. Peak and plateau pressures were high (32-38 cmH_2_O and 30-33 cmH_2_O, respectively), and lung compliance was decreased to 40-43 mL/cmH_2_0. Immediate point-of-care ultrasound (POCUS) showed right apical barcode signs (on M-mode ultrasound, the entire image shows parallel horizontal lines from top to bottom, resembling a barcode, a pathognomonic finding of pneumothorax), and the patient was subsequently diagnosed with tension pneumothorax. Immediate chest decompression with a right needle thoracostomy was performed, with a gush of air coming out. Following that, a chest tube was inserted. Blood pressure and oxygen saturation (SpO_2_) improved immediately, the chest drain was bubbling, and there was residual pneumothorax (Figure 2), which improved with negative pressure suctioning. Over the next 24 hours, the patient improved. She was obeying commands, was off vasopressors, and was weaned from the ventilator and extubated. The chest drain was removed on day three, and she was transferred to the ward, from where she was later discharged home. Follow-up in the outpatient clinic did not reveal any abnormalities.

## Discussion

Endobronchial intubation in the perioperative period is a preventable incident [1]. Endobronchial intubation is corrected by pulling the endotracheal tube, and the occurrence of tension pneumothorax is not reported in the literature. Endobronchial intubation accounts for 2% in the USA, in respiratory claims in adults, and 4% in children [2]. Up to 80% of endobronchial intubations are detected during maintenance of anesthesia with oxygen desaturation, commonly with a Ring-Adair-Elwyn (RAE) tube. One-third of the cases occur during positioning of the patient and in head and neck surgical patients [3]. Endobronchial intubation causes lung collapse and has increased chances of unplanned ICU admissions. Most of the time, it is difficult to appreciate the symmetry of chest movements, and no changes in capnography make it difficult to diagnose, leading to a delay in diagnosis of endobronchial intubation.

The majority of cases of endobronchial intubation can be prevented by checking the level of endotracheal tube insertion and oxygen desaturation at any stage of anesthesia, considering the possibility of endobronchial intubation [1]. Timely chest auscultation and EtCO_2_ monitoring are also helpful. Flexible bronchoscopy is the ideal and confirmatory way to diagnose abnormal endobronchial intubation. It also definitely guides the pulling and positioning of the endotracheal tube. Flexible bronchoscopy is widely available and easy to perform, and one can visualize the normal or abnormal position of ETT; as in this case, if it is in the right main bronchus under vision, it can be pulled above the carina [4]. More recently, POCUS is used with more accuracy to detect endobronchial intubation, causing lung atelectasis and collapse by the absence of unilateral lung sliding and the presence of lung pulse [5].

In our patient, after correction of endobronchial intubation, he developed tension pneumothorax, which was diagnosed by POCUS and initially managed with needle decompression and then required chest drain insertion with the application of negative pressure suction. The most probable reason for the occurrence of tension pneumothorax in our patient may be a larger tidal volume and pressure from ventilation with an Ambu bag while trying to open the collapsed lung, causing injury to the contralateral normal lung, resulting in barotrauma and further continuation of ventilation, leading to tension pneumothorax.

Ambu ventilation or volume-controlled ventilation has a high risk for volutrauma, barotrauma, and biotrauma, causing ventilation-associated injuries, longer intensive care stays, and higher mortality. Hence, it is recommended to ventilate these patients with lung-protective ventilation. In lung-protective ventilation, lower tidal volumes are used, plateau pressure is kept lower than 30 cm of H_2_O, with incremental increase in PEEP to keep the alveoli open while preventing ventilator-associated lung injury by reducing the overdistension (volutruama) and high pressure (barotrauma) [6]. When a ventilated patient desaturates either in the operating theatre or the intensive care units, one should immediately look for the aetiology and correct it quickly. This can be done swiftly and properly by using the “DOPE-R” mnemonic or algorithm. Here, D is detecting the tube displacement; O is airway obstruction; P is for developing pneumothorax; E is dictating equipment failure; and, in ventilated patients, high-dose fentanyl, especially when given rapidly, can result in chest wall rigidity, and hence R [7].

Recently, the ATLS® (Adult Trauma Life Support) guidelines recommend that the decompression for tension pneumothorax should be done at the fifth intercostal space anterior to the mid-axillary line, instead of the second intercostal space and midclavicular line [8]. However, the anterior approach for chest drain insertion is not inferior to the lateral approach in treating pneumothorax. The evidence suggests that the fourth-fifth intercostal space anterior to the mid-axillary line has the lowest predicted failure rate in multiple populations [9].

There are various causes of intraoperative desaturation, which can be easily detected and managed by following the “COVER” ABCD algorithm. Here, C stands for cardiovascular collapse, O is the oxygen supply issues, V is ventilation issues, E is endotracheal tube issues, and R stands for reviewing the monitor, equipment, and patients' review [10].

The concluding line from our case is that endobronchial intubation is a preventable event that can be detected by a high index of suspicion during intraoperative desaturation. After pulling or adjusting the endotracheal tube under vision with flexible bronchoscopy, lung protective ventilation should be used for opening of the collapsed lung to avoid barotrauma to the normal lung that can develop into life-threatening tension pneumothorax.

## Conclusions

Oxygen desaturation at any stage of anesthesia should raise suspicion for endobronchial intubation. Apart from clinical examination, the use of flexible bronchoscopy allows for the early and accurate diagnosis of endotracheal tube (ETT) displacement into the right main bronchus while providing vision for the ETT to be adjusted back into the trachea. Once the ETT is repositioned into the trachea, attempting to reopen the left collapsed lung with aggressive ventilation can cause right-sided pneumothorax and tension pneumothorax. Meticulous care should be taken to avoid endobronchial intubation. Following the repositioning of the endobronchial intubation, ventilation should be with lung-protective ventilation. Aggressive ventilation can damage the normal lung, causing pneumothorax.
